# Analyzing Many‐Body Charge Transfer Effects With the Fragment Molecular Orbital Method

**DOI:** 10.1002/jcc.70128

**Published:** 2025-05-15

**Authors:** Dmitri G. Fedorov

**Affiliations:** ^1^ Materials DX Research Center (MDX), National Institute of Advanced Industrial Science and Technology (AIST) Tsukuba Japan

**Keywords:** charge transfer, decomposition analysis, EDA, FMO, interaction, many‐body

## Abstract

A many‐body expansion of charge transfer (CT) energies is developed for the fragment molecular orbital method. It is applied to decouple CT and mix terms in interaction energy decomposition analyses. Many‐body charge transfer is graphically illustrated in the form of frontier orbital diagrams. The contribution of CT to molecular interactions is elucidated in the application of the method to water clusters, solvated ions, and polypeptide motifs.

## Graphical abstract

A many‐body expansion of the charge transfer energy has been developed for the fragment molecular orbital method and applied to a polypeptide motifs, water, and solvated ions. Many‐body CT is rationalized as a schematic interaction of molecular orbitals.

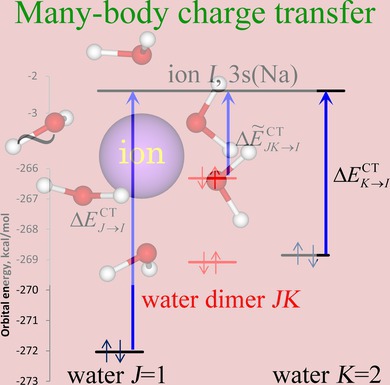

## 1 Introduction

Molecular interactions are of paramount importance to understanding chemical and physical phenomena [1]. One of the early approaches for quantifying interactions is the Kitaura–Morokuma (KM) [2, 3] energy decomposition analysis (EDA), followed by many other methods [4, 5, 6, 7, 8, 9, 10, 11, 12, 13, 14]. Fragmentation techniques [15, 16, 17, 18, 19, 20, 21, 22, 23, 24, 25] reduce the cost of quantum‐mechanical (QM) calculations, providing a convenient and efficient platform for evaluating interactions, which can be visualized using electron density [26].

Many but not all [24, 27, 28] of fragment‐based methods use a many‐body expansion [29, 30, 31]. Many‐body effects on molecular interactions have attracted steady interest over many years [32, 33, 34, 35, 36]. Frontier orbitals [37] can be used to rationalize reactivity [38] related to charge transfer and compute chemical hardness in conceptual density functional theory (DFT) [39].

The fragment molecular orbital (FMO) method [40, 41, 42, 43, 44] is an efficient QM approach for calculating large molecular systems. FMO delivers pair interaction energies that can be decomposed into physical components using several groups of methods: (a) a many‐body expansion of KM‐EDA integrated into FMO*n* [45, 46, 47, 48, 49], known as EDA*n* (EDA2 is also called the pair interaction EDA, abbreviated as PIEDA), (b) local molecular orbital based decompositions [50, 51], and (c) partition analyses [52, 53, 54]. An important term in these analyses is the solvent screening [55]. Interactions can be visualized with the non‐covalent interaction index [56].

The interaction analyses based on FMO have been used in a variety of biochemical [57, 58, 59, 60] and inorganic [61, 62, 63] applications, providing important insight into molecular binding and catalysis. By identifying hot spots, a rational drug or material design can be performed, improving drug efficacy [64, 65].

Polarization in the FMO framework may be viewed as an intrafragment 1‐body charge transfer caused by an external embedding potential, whereas this work deals with interfragment 2‐and 3‐body charge transfer.

In this work, a scheme for obtaining *n*‐body charge transfer energies in the framework of FMO is proposed and integrated into EDA*n* (*n* = 2 and 3), allowing for a separation of CT and mix terms. The mix term is the remainder of interaction couplings left over after other contributions are separated. The scheme is developed for two different embeddings: a fixed embedding due to isolated fragments can be used for a direct comparison to KM‐EDA, and a fully relaxed polarizable embedding that is the default way of doing FMO calculations.

## 2 Methodology

### 2.1 Introduction to FMO

In FMO, a molecular system is divided into *N* fragments. Their QM calculations can be done (a) without an embedding (the isolated state, denoted by 0), (b) with a fixed embedding from the electron densities of isolated fragments (the polarized state PL0), and (c) with a self‐consistently polarized embedding (the PL state). The PL state is most commonly used in applications of FMO, whereas the 0 and PL0 states are used in some analyses, mainly to define polarization energies [45, 66, 67].

The energy in the three‐body FMO3 is obtained as [68]

(1) $$E=\sum_{I=1}^{N}E_{I}^{\prime }+\sum_{I>J}^{N}\Delta E_{\mathrm{IJ}}+\sum_{I>J>K}^{N}\Delta E_{\mathrm{IJK}}$$

and the corresponding two‐body FMO2 energy is computed by neglecting the last term in Equation (1). $E_{I}^{\prime }$ is the internal energy of polarized fragment *I*, $\Delta E_{\mathrm{IJ}}$ is the pair interaction energy (PIE) for fragments *I* and *J*, whereas $\Delta E_{\mathrm{IJK}}$ is the three‐body coupling of *IJ*, *IK*, and *JK* PIEs.

The PL0 state is used to make a direct comparison of FMO*n*‐based analyses of interactions (EDA*n*) with KM‐EDA [2], because both are formulated for the binding in a complex relative to isolated states of molecules forming it. KM‐EDA has various limitations (neither density functional theory (DFT) nor solvent models may be used, spherical atomic orbitals are not supported etc.), whereas EDA*n* is much more general and removes all of these limitations.

A full comparison of EDA2 with KM‐EDA requires the use of all three states (0, PL0, and PL) in EDA2 in order to define the many‐body polarization (whose values are identical in the two approaches [45]). EDA*n* is an *n*‐body approximation to KM‐EDA, the latter being an exact decomposition of binding energies. Originally [45], the PL0 state was developed only for FMO2, and in this work it is extended to FMO3.

### 2.2 Definition of Charge Transfer Energies for the PL State

In the original EDA*n* [45, 48], CT and higher order MIX terms are computed as a sum CT + MIX. On the other hand, KM‐EDA2 provides a way for their decoupling. In this work, the KM‐EDA recipe for CT is modified and integrated into the PIEDA scheme.

In EDA2 applied to second‐order Møller–Plesset perturbation theory (MP2), PIE is decomposed into electrostatic (ES), exchange‐repulsion (EX), CT + MIX, remainder correlation (RC) plus dispersion (DI), and solvent (SOLV) screening terms.

(2) $$\Delta E_{\mathrm{IJ}}=\Delta E_{\mathrm{IJ}}^{\mathrm{ES}}+\Delta E_{\mathrm{IJ}}^{\mathrm{EX}}+\Delta E_{\mathrm{IJ}}^{\mathrm{CT}+\mathrm{MIX}}+\Delta E_{\mathrm{IJ}}^{\mathrm{RC}+\mathrm{DI}}+\Delta E_{\mathrm{IJ}}^{\text{SOLV}}$$

In HF, there is no RC and $\Delta E_{\mathrm{IJ}}^{\mathrm{RC}+\mathrm{DI}}=\Delta E_{\mathrm{IJ}}^{\mathrm{DI}}$, where DI is obtained using some empirical model [69]. In DFT, DI is obtained likewise, and RC is computed as the correlation functional contribution [47].

(3) $$\Delta E_{\mathrm{IJ}}^{\mathrm{RC}+\mathrm{DI}}=\Delta E_{\mathrm{IJ}}^{\mathrm{RC}}+\Delta E_{\mathrm{IJ}}^{\mathrm{DI}}$$

In practice, $\Delta E_{\mathrm{IJ}}^{\mathrm{ES}}$, $\Delta E_{\mathrm{IJ}}^{\mathrm{EX}}$, $\Delta E_{\mathrm{IJ}}^{\mathrm{RC}+\mathrm{DI}}$ and $\Delta E_{\mathrm{IJ}}^{\text{SOLV}}$ are explicitly calculated, whereas the remainder is obtained as

(4) $$\Delta E_{\mathrm{IJ}}^{\mathrm{CT}+\mathrm{MIX}}=\Delta E_{\mathrm{IJ}}-\left(\Delta E_{\mathrm{IJ}}^{\mathrm{ES}}+\Delta E_{\mathrm{IJ}}^{\mathrm{EX}}+\Delta E_{\mathrm{IJ}}^{\mathrm{RC}+\mathrm{DI}}+\Delta E_{\mathrm{IJ}}^{\text{SOLV}}\right)$$

In the new development of this work, asymmetric pairwise charge transfers are defined. The energy $\Delta E_{I\to J}^{\mathrm{CT}}$ of CT from *I* to *J* is obtained by mixing occupied molecular orbitals of *I* with virtual orbitals of *J*, in a constrained SCF calculation [2, 3]. In this work it was found that to do that in FMO, instead of the regular projection operator [70] for dimer *IJ*, the following form should be used,

(5) $$P^{\mathrm{IJ}}=P^{I}⊕P^{J}$$

This is a critical step for systems with detached covalent bonds, because the regular projection operator for unconstrained SCF of dimer *IJ*, computed without the block form of Equation (5), results in a large potential acting on monomer *I* from projected‐out orbitals in monomer *J* and vice versa. In unconstrained calculations, a full orbital relaxation minimizes the effect of the projection, but in constrained calculations it does not happen. A further justification for the form of the projection is given in SI.

The total energy of pairwise charge transfer in dimer *IJ* is

(6) $$\Delta E_{\mathrm{IJ}}^{\mathrm{CT}}=\Delta E_{I\to J}^{\mathrm{CT}}+\Delta E_{J\to I}^{\mathrm{CT}}$$

The decomposition in MP2 becomes

(7) $$\Delta E_{\mathrm{IJ}}=\Delta E_{\mathrm{IJ}}^{\mathrm{ES}}+\Delta E_{\mathrm{IJ}}^{\mathrm{EX}}+\Delta E_{\mathrm{IJ}}^{\mathrm{CT}}+\Delta E_{\mathrm{IJ}}^{\mathrm{MIX}}+\Delta E_{\mathrm{IJ}}^{\mathrm{RC}+\mathrm{DI}}+\Delta E_{\mathrm{IJ}}^{\text{SOLV}}$$

where the remainder MIX term is obtained as

(8) $$\Delta E_{\mathrm{IJ}}^{\mathrm{MIX}}=\Delta E_{\mathrm{IJ}}^{\mathrm{CT}+\mathrm{MIX}}-\Delta E_{\mathrm{IJ}}^{\mathrm{CT}}$$

In FMO3‐MP2, CT and MIX terms can be split, yielding the following:

(9) $$\begin{array}{l} \Delta E_{\mathrm{IJK}}=\Delta E_{\mathrm{IJK}}^{\mathrm{EX}}+\Delta E_{\mathrm{IJK}}^{\mathrm{CT}+\mathrm{MIX}}+\Delta E_{\mathrm{IJK}}^{\mathrm{RC}+\mathrm{DI}}+\Delta E_{\mathrm{IJK}}^{\text{SOLV}}\\ \;=\Delta E_{\mathrm{IJK}}^{\mathrm{EX}}+\Delta E_{\mathrm{IJK}}^{\mathrm{CT}}+\Delta E_{\mathrm{IJK}}^{\mathrm{MIX}}+\Delta E_{\mathrm{IJK}}^{\mathrm{RC}+\mathrm{DI}}+\Delta E_{\mathrm{IJK}}^{\text{SOLV}} \end{array}$$

ES and empirical DI models are pairwise additive (no three‐body contributions). On the other hand, EX is obtained from the exchange‐repulsion interaction between occupied MOs of *I*, *J*, and *K* in trimer *IJK*, including the effect of the delocalization, so that the three‐body EX term is not additive. For HF, $\Delta E_{\mathrm{IJK}}^{\mathrm{RC}+\mathrm{DI}}=0$ and for DFT, $\Delta E_{\mathrm{IJK}}^{\mathrm{RC}+\mathrm{DI}}=\Delta E_{\mathrm{IJK}}^{\mathrm{RC}}$.

In the multi‐fragment extension of KM‐EDA [3], CT energy is computed by allowing the mixing of occupied orbitals of a fragment *I* with virtual orbitals of all other fragments (*J* ≠ *I*). Initially, the same strategy was also attempted in the FMO framework. It was found, however, that the CT mechanism in multi‐fragment KM‐EDA is often unstable (SCF diverges). The origin of this instability was identified to be the coupling of virtual orbitals of multiple isolated fragments that causes an appearance of low‐lying spurious virtual MOs with an energy similar to the energy of occupied orbitals, resulting in a metallic type of divergence in SCF. For the original KM‐EDA scheme [2] applied to 2 fragments, there is only one set of virtual orbitals and the problem does not arise. Another problem of the multi‐fragment KM‐EDA3 is that the CT from occupied orbitals of *I*,*J* to virtual orbitals of other fragments is not defined.

Therefore, a different strategy was adopted for three‐body terms in EDA3. The CT recipe of KM‐EDA was applied to all combinations of the type: dimer *IJ* and monomer *K*, treated in KM‐EDA as two subsystems (*IJ* and *K*). This yields $\Delta {\tilde{E}}_{K\to \mathrm{IJ}}^{\mathrm{CT}}$ terms (occupied *K* to virtual *IJ*) and $\Delta {\tilde{E}}_{\mathrm{IJ}\to K}^{\mathrm{CT}}$ terms (occupied *IJ* to virtual *K*). CT values in HF and MP2 are identical, because the electron correlation contribution in MP2 is a separate term RC + DI.

The obtained $\Delta {\tilde{E}}_{K\to \mathrm{IJ}}^{\mathrm{CT}}$ and $\Delta {\tilde{E}}_{\mathrm{IJ}\to K}^{\mathrm{CT}}$ terms describe CT effects within trimer *IJK*, combining two and three‐body contributions. For analysis, it is useful to subtract two‐body values and define a three‐body CT as,

(10) $$\Delta E_{K\to \mathrm{IJ}}^{\mathrm{CT}}=\Delta {\tilde{E}}_{K\to \mathrm{IJ}}^{\mathrm{CT}}-\Delta E_{K\to I}^{\mathrm{CT}}-\Delta E_{K\to J}^{\mathrm{CT}}$$

(11) $$\Delta E_{\mathrm{IJ}\to K}^{\mathrm{CT}}=\Delta {\tilde{E}}_{\mathrm{IJ}\to K}^{\mathrm{CT}}-\Delta E_{I\to K}^{\mathrm{CT}}-\Delta E_{J\to K}^{\mathrm{CT}}$$

The projection operator for constrained SCF used for computing *IJ*→*K* and *K* → *IJ* terms in trimer *IJK* is obtained similarly to the dimer case.

(12) $$P^{\mathrm{IJK}}=P^{\mathrm{IJ}}⊕P^{K}$$

The total three‐body CT energy in trimer *IJK* is

(13) $$\Delta E_{\mathrm{IJK}}^{\mathrm{CT}}=\Delta E_{I\to \mathrm{JK}}^{\mathrm{CT}}+\Delta E_{J\to \mathrm{IK}}^{\mathrm{CT}}+\Delta E_{K\to \mathrm{IJ}}^{\mathrm{CT}}+\Delta E_{\mathrm{IJ}\to K}^{\mathrm{CT}}+\Delta E_{\mathrm{IK}\to J}^{\mathrm{CT}}+\Delta E_{\mathrm{JK}\to I}^{\mathrm{CT}}$$

The three‐body mixing term is defined as

(14) $$\Delta E_{\mathrm{IJK}}^{\mathrm{MIX}}=\Delta E_{\mathrm{IJK}}^{\mathrm{CT}+\mathrm{MIX}}-\Delta E_{\mathrm{IJK}}^{\mathrm{CT}}$$

where $\Delta E_{\mathrm{IJK}}^{\mathrm{CT}+\mathrm{MIX}}$ is obtained by subtracting all other components in Equation (9) from $\Delta E_{\mathrm{IJK}}$.

It may be expected that an $\Delta E_{I\to \mathrm{JK}}^{\mathrm{CT}}$ ($\Delta E_{\mathrm{JK}\to I}^{\mathrm{CT}}$) term is substantial if (a) *J* and *K* are nearly in resonance, that is, if virtual (occupied) orbital energies of *J* and *K* are similar and (b) the coupling is large, which is strongly affected by the separation between *J* and *K*.

The three‐body coupling of solute (or explicit solvent) fragment *I* with solvent fragments *J* and *K* can explain large three‐body effects observed in pure solvents [71] and solvated ions [72], attributed to the near resonance of virtual orbitals of water. Especially when diffuse functions are present, low virtual orbitals of all fragments tend to be nearly degenerate [73], promoting charge transfer and diminishing the accuracy of FMO*n*, with CT truncated at *n*‐body level.

All pairwise CT terms are negative (attractive) because they are obtained in SCF as an energy minimization, so the energy can only decrease when occupied and virtual orbitals are allowed to mix. Differential three‐body CT energies can be both negative and positive.

### 2.3 Definition of Charge Transfer Energy for the PL0 State

The PL0 state is used to obtain interactions between isolated (nonpolarized) fragments, and the fixed embedding of fragments is computed from the density of isolated fragments. The embedding potential of any fragment *X* (*X* = *I*, *IJ*, or *IJK*) is computed using the electron densities (or charges) of all other fragments L≠X.

The peculiarity of PL0 is that in a dimer *IJ*, isolated (nonpolarized) fragments *I* and *J* are polarized, and their pair interaction includes a polarization contribution. The decomposition of the interaction energy between *I* and *J* in EDA2/PL0 for MP2 is [45]

(15) $$\begin{array}{c} \Delta E_{\mathrm{IJ}}^{\mathrm{PL}0}=\Delta E_{\mathrm{IJ}}^{\mathrm{ES},0}+\Delta E_{\mathrm{IJ}}^{\mathrm{EX},0}+\Delta E_{\mathrm{IJ}}^{\mathrm{CT},0}+\Delta E_{\mathrm{IJ}}^{\mathrm{MIX},0}\\ \;+\Delta E_{\mathrm{IJ}}^{\mathrm{RC}0+\mathrm{DI}0}+\Delta E_{\mathrm{IJ}}^{\mathrm{POL}0}+\Delta E_{\mathrm{IJ}}^{\text{SOLV}0} \end{array}$$

where the polarization $\Delta E_{\mathrm{IJ}}^{\mathrm{POL}0}$ is obtained [45] as the sum of the polarization energies of the two fragments $\Delta E_{I}^{\mathrm{POL}0}$ and $\Delta E_{J}^{\mathrm{POL}0}$,

(16) $$\Delta E_{\mathrm{IJ}}^{\mathrm{POL}0}=\Delta E_{I}^{\mathrm{POL}0}+\Delta E_{J}^{\mathrm{POL}0}$$

The exchange‐repulsion contribution $\Delta E_{\mathrm{IJ}}^{\mathrm{EX},0}$ is computed between the molecular orbitals of isolated fragments *I* and *J*. The charge transfer term $\Delta E_{\mathrm{IJ}}^{\mathrm{CT},0}$ between isolated fragments *I* and *J* is obtained using their molecular orbitals by constraining SCF as described above for the PL state. Likewise, for trimers,

(17) $$\Delta E_{\mathrm{IJK}}^{\mathrm{PL}0}=\Delta E_{\mathrm{IJK}}^{\mathrm{EX},0}+\Delta E_{\mathrm{IJK}}^{\mathrm{CT},0}+\Delta E_{\mathrm{IJK}}^{\mathrm{MIX},0}+\Delta E_{\mathrm{IJK}}^{\mathrm{RC}0+\mathrm{DI}0}+\Delta E_{\mathrm{IJK}}^{\mathrm{POL}0}+\Delta E_{\mathrm{IJK}}^{\text{SOLV}0}$$

where the polarization term in trimer *IJK* is (see SI for a justification)

(18) $$\begin{array}{l} \Delta E_{\mathrm{IJK}}^{\mathrm{POL}0}=\Delta E_{I}^{\mathrm{POL}0}+\Delta E_{J}^{\mathrm{POL}0}+\Delta E_{K}^{\mathrm{POL}0}-\left(\Delta E_{\mathrm{IJ}}^{\mathrm{POL}0}+\Delta E_{\mathrm{IK}}^{\mathrm{POL}0}+\Delta E_{\mathrm{JK}}^{\mathrm{POL}0}\right)\\ \;=-\left(\Delta E_{I}^{\mathrm{POL}0}+\Delta E_{J}^{\mathrm{POL}0}+\Delta E_{K}^{\mathrm{POL}0}\right) \end{array}$$

The charge transfer term $\Delta E_{\mathrm{IJK}}^{\mathrm{CT},0}$ between isolated fragments *I*, *J*, and *K* is obtained using their molecular orbitals by constraining SCF as described above for the PL state, with one important change: for consistency, in PL0 it is necessary to compute three‐body CT in *IJK* between dimer *IJ*, computed without embedding, with isolated monomer *K*. In contrast, for the PL state three‐body CT is computed between polarized dimer *IJ* and monomer *K*.

The rapid convergence of the many‐body expansion of the electron density [41, 74] suggests that the expansion of the CT may also converge. A different many‐body expansion of the electron density in the Taylor series is used in the density‐functional tight‐binding (DFTB) methods [75], in which the third‐order DFTB3 method is commonly used.

### 2.4 Definition of Total Interaction Components

The total values for each energy component *A* in EDA/PL0 can be summed as (*A* = ES, EX, CT, RC, and MIX).

(19) $$\Delta E^{A,0}=\sum_{I>J}^{N}\Delta E_{\mathrm{IJ}}^{A,0}$$

and likewise the total interaction ΔE is computed by summing $\Delta E_{\mathrm{IJ}}$. Similarly, the components in EDA/PL can be obtained.

(20) $$\Delta E^{A}=\sum_{I>J}^{N}\Delta E_{\mathrm{IJ}}^{A}$$

The many‐body treatment of CT in FMO based on occupied and virtual orbital mixing may be compared to the many‐body expansion of (a) frontier orbital energies in the Koopmans' approach to obtaining ionization potentials and electron affinities [76] and (b) excited state energies [77, 78], both of which are applied to systems containing a single chromophore with a weak coupling to other fragments. The CT method proposed here is composite, covering the whole orbital space in all fragments.

### 2.5 CT Analysis for Auxiliary Polarization Methods

For molecular clusters, there is an accurate scheme of damped point charges [79] used in the embedding, employed in FMO for large basis sets. For fragments with covalent boundaries, a different methodology is used, an auxiliary polarization (AP) [80]. Recently, AP was extended to use caps (C) for covalent bonds on fragment boundaries in the APC method [73, 81].

APC requires three FMO calculations, performed with a specific combination of an embedding (*V*) or no embedding (0) and two basis sets (a smaller basis BS1 and a larger basis BS2). PIE in AP(C) is obtained as

(21) $$\Delta E_{\mathrm{IJ}}=\Delta E_{\mathrm{IJ}}\left(V,\mathrm{BS}1\right)-\Delta E_{\mathrm{IJ}}\left(0,\mathrm{BS}1\right)+\Delta E_{\mathrm{IJ}}\left(0,\mathrm{BS}2\right)$$

$\Delta E_{\mathrm{IJ}}$ in the three terms of Equation (21) are decomposed in Equation (7). The composite values of CT in EDA*n*/APC are computed as

(22) $$\Delta E_{I\to J}^{\mathrm{CT}}=\Delta E_{I\to J}^{\mathrm{CT}}\left(V,\mathrm{BS}1\right)-\Delta E_{I\to J}^{\mathrm{CT}}\left(0,\mathrm{BS}1\right)+\Delta E_{I\to J}^{\mathrm{CT}}\left(0,\mathrm{BS}2\right)$$

(23) $$\Delta E_{I\to \mathrm{JK}}^{\mathrm{CT}}=\Delta E_{I\to \mathrm{JK}}^{\mathrm{CT}}\left(V,\mathrm{BS}1\right)-\Delta E_{I\to \mathrm{JK}}^{\mathrm{CT}}\left(0,\mathrm{BS}1\right)+\Delta E_{I\to \mathrm{JK}}^{\mathrm{CT}}\left(0,\mathrm{BS}2\right)$$

The CT calculation proposed in this work can be done in APC for unconnected fragment pairs (pairs without covalent bonds between them) only for the PL state.

## 3 Computational Details

In all calculations, FMO implemented [82] in GAMESS [83, 84] was used, parallelized with the generalized distributed data interface [85, 86]. KM‐EDA has the limitation that Cartesian atomic orbitals (AO) should be used (ISPHER = −1). For EDA*n*, both Cartesian and spherical (ISPHER = 1) AO may be used. This was enabled by using monomer MOs obtained with spherical AOs and transforming Fock matrices to this MO basis according to the general recipe of KM‐EDA.

The point charge approximation with a damping prefactor [79] was employed for computing the embedding for diffuse basis sets. Facio was used for making input files [87]. In DFT, the CAM‐B3LYP functional was used with the default Lebedev grid.

The structures of water clusters and solvated ions were optimized at the level of DFT/6–311++G**/D3(BJ) similarly to the previous study [48]. The structures of the α‐helix and β‐turn of a capped polypeptide (ALA)_10_ were optimized using FMO2‐DFTB3 and the polarizable continuum model (PCM) [88] with 3ob parameters [89] and D3(BJ) dispersion [69].

In the calculations of ions, the first solvation shell containing 6 water molecules was immersed in a continuum solvent (water) described with the solvation model density (SMD < 1 >) [90, 91]. For +2 and +3 ions, the RSTRCT option in $SCF was used in constrained CT calculations of dimers and trimers to achieve convergence. Molecular clusters were divided as 1 molecule per fragment (unless stated otherwise), polypeptides as 1 conventional residue [92] per fragment.

## 4 Results and Discussion

### 4.1 Validation of CT for Isolated Fragments

In order to compare EDA2 with KM‐EDA, HF calculations are done in the gas phase using Cartesian AOs. The results are shown in Tables 1, S1, and S2.

**TABLE 1:** Comparison of EDA2 components (kcal/mol) versus full KM‐EDA for (H_2_O)_2_ at the level of HF/6–311++G**.

Method	ESP^a^	ES	EX	CT(*I* → *J*)^b^	CT(*J* → *I*)^b^	MIX^c^
EDA2/PL0	damp‐pc	−12.599	11.862	−0.781	−2.025	−0.248 (−0.052)
EDA2/PL0	density	−12.599	11.862	−0.781	−2.025	1.059 (1.170)
EDA2/PL	damp‐pc	−13.519	11.366	−0.473	−2.018	0.129
EDA2/PL	density	−16.478	13.914	−1.489	−3.293	1.093
EDA2/AP/PL	density	−14.619	11.950	−0.779	−1.962	0.234
KM‐EDA	density	−12.599	11.862	−0.781	−2.025	1.170

^a^ Electrostatic potential: damped point charges (damp‐pc) or electron density based.

^b^ *I* = 1 and *J* = 2 number the two water molecules in (H_2_O)_2_.

^c^ In parentheses, the value corrected for polarization coupling (PL⋅PL) is shown.

For the PL0 state, comparing EDA2 with KM‐EDA, an exact agreement is observed for all terms including CT (the main subject of this work) except for MIX. The MIX term ($\Delta E_{\mathrm{IJ}}^{\mathrm{MIX},0}$) in EDA2 includes the effect of the polarization coupling (the PL⋅PL term [45]), whereas in KM‐EDA the PL⋅PL term is separate. The exact agreement of EDA2 to KM‐EDA is achieved (1.170 kcal/mol) when the PL⋅PL effect is subtracted provided that the exact ESP is used (obtained from the density). For the damped ESP, the MIX term in EDA2 differs from KM‐EDA because the polarization is affected by the form of the embedding.

For the PL state in EDA2, all terms differ from KM‐EDA because they are computed for polarized fragments (compared with isolated fragments in KM‐EDA). A closer agreement is observed for the damped ESP, consistent with the general finding that the PL state of molecular clusters for diffuse basis sets is better described with this ESP. [79] In the rest of this work, the damped ESP is used for all molecular clusters.

When AP is used for the PL state, the results are much closer to the damped ESP energies for the same PL state, as expected because these two approaches differ only in the form of the embedding potential affecting the polarization. AP largely alleviates the problem of the density‐based ESP used for large basis sets.

The effect of using Cartesian vs. spherical AOs is shown in Table S3. It is negligible (0.01 kcal/mol or less). The technique to remove spherical contaminants developed in this work is appropriate. In the rest of this work, spherical AOs are used.

### 4.2 Polypeptide Motifs

**FIGURE 1:**
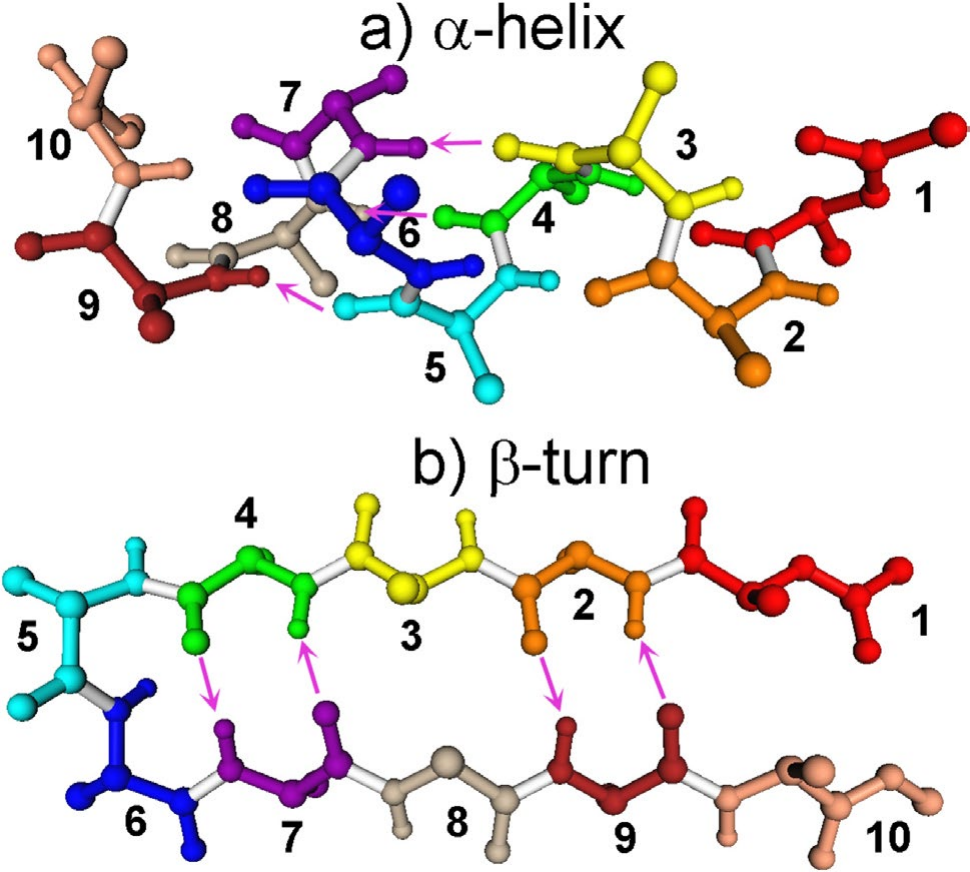
Fragments in the (a) α‐helix and (b) β‐turn of (ALA)_10_. Hydrogen atoms other than those forming hydrogen bonds (pink arrows from CO to NH) are not shown.

The CT method can be applied to systems with covalent fragment boundaries that are not possible to treat in KM‐EDA. Two isomers of (ALA)_10_ were computed: the α‐helix and β‐turn (Figure 1).

The results are shown in Table 2. The α‐helix has a period of 4, so inner hydrogen bonds are found between *I*,*I* + 4 residues. For the α‐helix, there is a unidirectional charge transfer from CO (residue *J* with the smaller ID in a pair *IJ*, *J* = 3, 4, or 5) to HN (residue *I* with the larger ID, *I* = 7, 8, or 9), so that $\Delta E_{J\to I}^{\mathrm{CT}}$ is the dominant term, although the back donation $\Delta E_{I\to J}^{\mathrm{CT}}$ is not negligible. For the β‐turn, the charge transfer is bidirectional, because there are two hydrogen bonds per residue pair in the opposite order of CO … HN. $\Delta E_{J\to I}^{\mathrm{CT}}$ and $\Delta E_{I\to J}^{\mathrm{CT}}$ have comparable magnitudes.

**TABLE 2:** Charge transfer energies (kcal/mol) in representative inner hydrogen bonds of the α‐helix and β‐turn of (ALA)_10_ at the level of DFT/APC/SMD.^a^

		$\Delta E_{I\to J}^{\mathrm{CT}}$		$\Delta E_{J\to I}^{\mathrm{CT}}$		$\Delta E_{\mathrm{IJ}}^{\mathrm{MIX}}$	
isomer	*I*,*J*
6‐31G**	6–311++G**	6‐31G**	6–311++G**	6‐31G**	6–311++G**
α‐helix	7,3	−0.46	−0.77	−3.08	−2.35	−2.84	−2.23
α‐helix	8,4	−0.46	−0.85	−3.02	−2.09	−2.27	−1.64
α‐helix	9,5	−0.30	−0.56	−2.45	−1.62	−1.82	−1.20
β‐turn	7,4	−2.74	−2.44	−2.42	−1.98	−3.45	−2.19
β‐turn	9,2	−2.56	−2.28	−2.52	−2.27	−2.51	−1.12

^a^ See Figure 1. In *IJ* pairs of the α‐helix, *I* has NH and *J* has CO.

Overall, the basis set effect appears to be: (a) a weakening of CT in CO → HN for short contacts (hydrogen bonds), and (b) a strengthening of the backward charge transfer in the α‐helix. The MIX term is substantial for polypeptides, and it is smaller for the larger basis set.

### 4.3 Many‐Body CT

**FIGURE 2:**
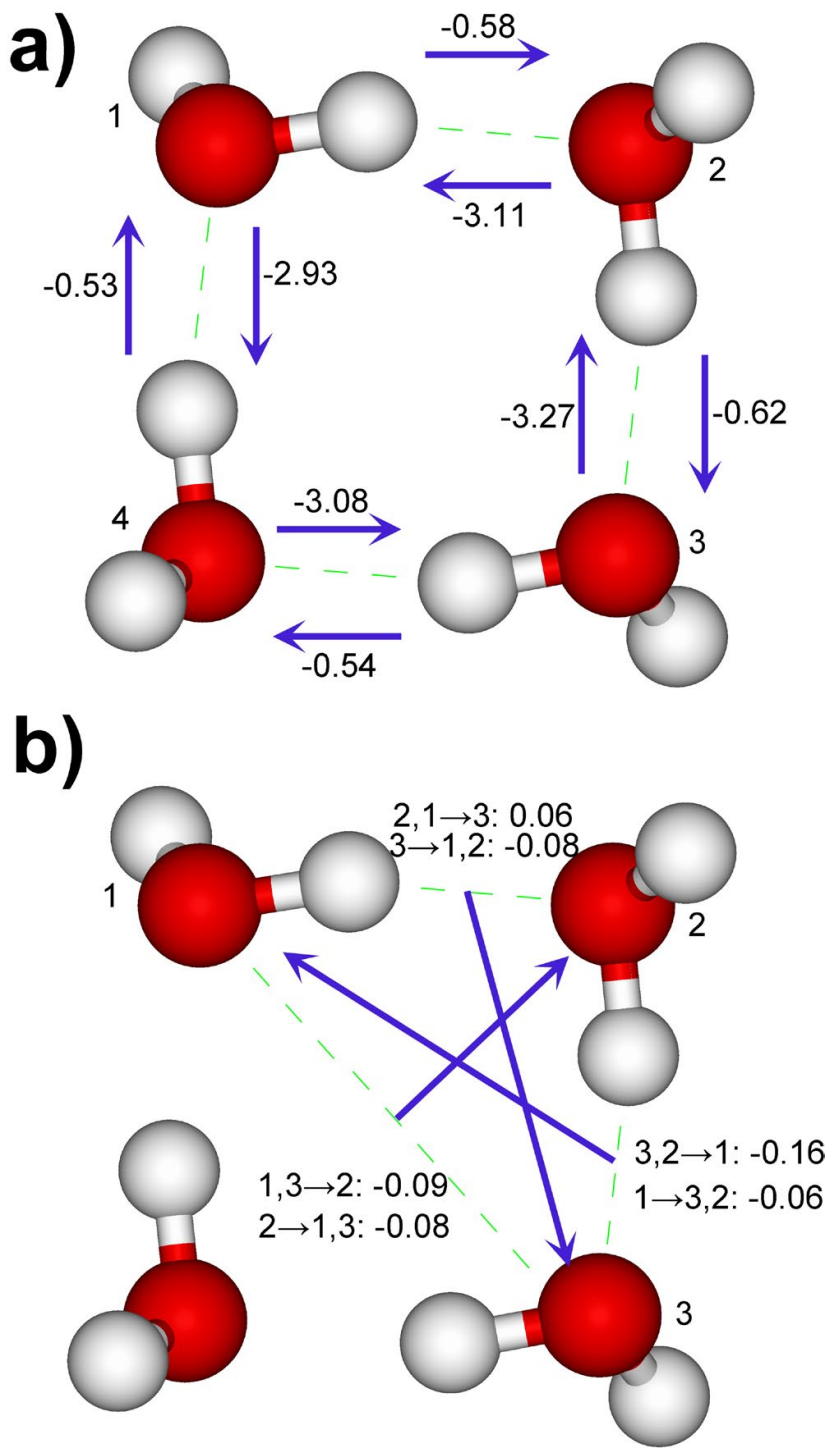
Charge transfer energies in kcal/mol, (a) $\Delta E_{I\to J}^{\mathrm{CT}}$ (for adjacent pairs) and (b) $\Delta E_{\mathrm{IJ}\to K}^{\mathrm{CT}}$ and $\Delta E_{K\to \mathrm{IJ}}^{\mathrm{CT}}$ (for one trimer 1, 2, 3) in (H_2_O)_4_ at the level of HF/6–311 + G**. For trimers, arrows are drawn from the middle point of *IJ* towards *K*. Other CT values are omitted for simplicity.

CT components for the PL state of (H_2_O)_4_ are shown in Figure 2. Pairwise MIX terms are less than 0.34 kcal/mol. Pairwise CT values are about −3.0 kcal/mol for the electron donation from the lone pair of O of one water to H of another water. There is also a backward charge donation with the energy of about −0.6 kcal/mol. The total energy of CT is about −3.6 kcal/mol per hydrogen bond. The CT energies for non‐adjacent pairs are not shown in Figure 2; they are about −0.04 … −0.02 for both forward and backward CT.

Three‐body charge transfer terms are small, on the order of 0.1 kcal/mol, much smaller than the two‐body values. The contributions of *IJ*→*K* and *K* → *IJ* are roughly of the same magnitude, so that the coupling of both occupied‐occupied‐virtual ($\Delta E_{\mathrm{IJ}\to K}^{\mathrm{CT}}$) and occupied‐virtual‐virtual ($\Delta E_{K\to \mathrm{IJ}}^{\mathrm{CT}}$) types are important.

The results for (H_2_O)_8_ (with 8 water molecules forming a cube) are presented in Table 3. For HF, there is no electron correlation and the RC term is 0. Three‐body effects are smaller than 6% of two‐body values. The MIX terms are systematically larger for the PL0 state, which is expected because the non‐polarized state of fragments deviates more from the electron state of dimers, and the difference accumulates as MIX coupling terms. For the PL state, MIX terms are small, whereas for the PL0 state, especially at the DFT level, MIX terms are large.

**TABLE 3:** Comparison of the total components of interactions^a^ (kcal/mol) in EDA*n* for (H_2_O)_8_.

*n*	Method	ES	EX	CT	MIX	RC	total
2	HF/PL0	−169.3	176.5	−47.3	−5.1	0.0	−45.2
2	HF/PL	−195.1	169.5	−39.7	2.0	0.0	−63.3
3	HF/PL0	−169.3	175.7	−46.8	−9.9	0.0	−50.3
3	HF/PL	−195.1	168.9	−42.1	1.4	0.0	−66.9
2	DFT/PL0	−177.2	195.1	−54.8	0.8	−42.0	−78.1
2	DFT/PL	−201.5	202.6	−48.6	−7.6	−41.5	−96.6
3	DFT/PL0	−177.2	236.0	−54.0	−47.6	−39.6	−82.4
3	DFT/PL	−201.5	200.8	−50.1	−9.5	−39.0	−99.3

^a^ The total values here are computed according to Equations (19) and (20) (6–311++G**).

Comparing HF and DFT, it is useful to consider the interfragment HOMO‐LUMO splitting among all fragment pairs [73], which is 15.1 and 10.9 eV for HF and DFT, respectively (for the PL state). This indicates that CT should be larger for DFT (the smaller gap), and indeed it is found that CT in DFT is larger by about 20% (comparing −42.1 and − 50.1 kcal/mol).

### 4.4 Solvated Ions (DFT)

**FIGURE 3:**
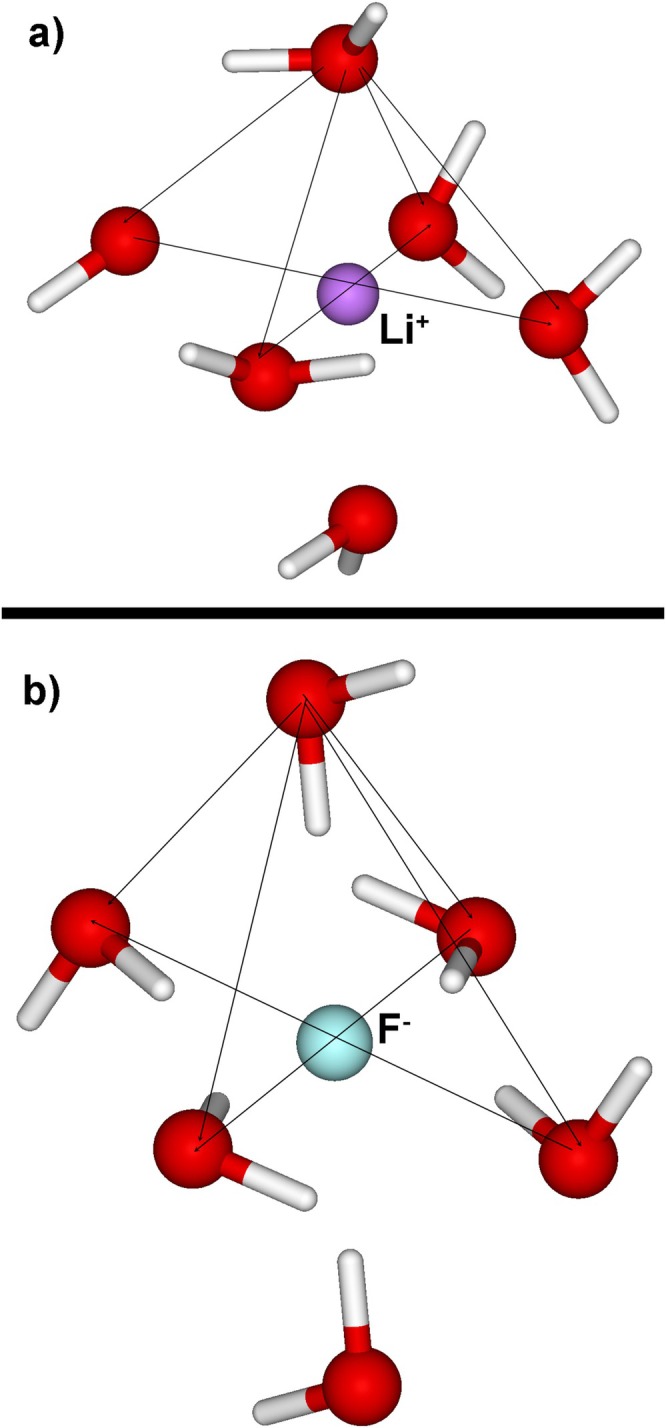
Structure of the solvated complexes for (a) Li^+^(H_2_O)_6_ and (b) F^−^(H_2_O)_6_. The upper prism of the distorted octahedron is demarcated with thin lines.

Many‐body CT energy effects for solvated ions *A*(H_2_O)_6_ (Figure 3) obtained in EDA3/PL calculations are presented in Tables 4 and S4 (more details are given in Tables S5 and S6). Each term is averaged over all water molecules; for example (denoting by *I* the fragment to which ion *A* is assigned),

(24) $$\Delta {\bar{E}}_{A\to 1\text{water}}^{\mathrm{CT}}=\frac{1}{6}\sum_{J=1}^{\text{water}}\Delta E_{I\to J}^{\mathrm{CT}}$$

(25) $$\Delta {\bar{E}}_{A\text{water}\to \text{water}}^{\mathrm{CT}}=\frac{1}{6\cdot 5}\sum_{J=1}^{\text{water}}\sum_{K\neq J}^{\;\text{water}}\Delta E_{\mathrm{IJ}\to K}^{\mathrm{CT}}$$

(26) $$\Delta {\bar{E}}_{A\to 2\text{waters}}^{\mathrm{CT}}=\frac{1\cdot 2}{6\cdot 5}\sum_{J=2}^{\text{water}}\sum_{K=1}^{J-1}\Delta E_{I\to \mathrm{JK}}^{\mathrm{CT}}$$

**TABLE 4:** Averaged many‐body charge transfer energies (kcal/mol) in *A*(H_2_O)_6_, DFT/6–311++G**.^a^

*A*	$\Delta {\bar{E}}_{A\to 1\text{water}}^{\mathrm{CT}}$	$\Delta {\bar{E}}_{1\text{water}\to A}^{\mathrm{CT}}$	$\Delta {\bar{E}}_{A\to 2\text{waters}}^{\mathrm{CT}}$	$\Delta {\bar{E}}_{2\text{waters}\to A}^{\mathrm{CT}}$	$\Delta {\bar{E}}_{A\text{water}\to \text{water}}^{\mathrm{CT}}$	$\Delta {\bar{E}}_{\text{water}\to A\text{water}}^{\mathrm{CT}}$
	2‐body	2‐body	3‐body	3‐body	3‐body	3‐body
Li^+^	−0.02 ± 0.00	−1.26 ± 0.14	0.00 ± 0.00	−0.49 ± 0.16	0.01 ± 0.01	−0.02 ± 0.03
Na^+^	−0.04 ± 0.01	−0.41 ± 0.08	−0.01 ± 0.01	−0.21 ± 0.06	0.00 ± 0.02	−0.02 ± 0.02
K^+^	−0.07 ± 0.01	−0.87 ± 0.06	0.00 ± 0.00	−0.08 ± 0.05	0.00 ± 0.00	0.00 ± 0.01
Be^2+^	0.00 ± 0.00	−10.68 ± 0.38	0.00 ± 0.00	0.55 ± 0.54	0.04 ± 0.08	1.13 ± 0.17
Mg^2+^	−0.03 ± 0.00	−3.10 ± 0.04	−0.01 ± 0.00	−1.01 ± 0.15	0.17 ± 0.04	−0.10 ± 0.05
Ca^2+^	−0.11 ± 0.01	−6.40 ± 0.17	0.00 ± 0.01	−2.11 ± 0.87	0.19 ± 0.03	0.16 ± 0.12
Al^3+^	−0.02 ± 0.00	−15.82 ± 0.11	−0.01 ± 0.00	−3.27 ± 1.02	0.56 ± 0.15	0.67 ± 0.25
F^−^	−3.45 ± 0.22	−0.08 ± 0.01	0.35 ± 0.11	−0.02 ± 0.02	0.13 ± 0.03	0.01 ± 0.02
Cl^−^	−3.25 ± 0.22	−0.15 ± 0.01	0.04 ± 0.08	−0.02 ± 0.01	0.08 ± 0.02	−0.03 ± 0.02
Br^−^	−2.92 ± 0.22	−0.19 ± 0.02	−0.06 ± 0.10	−0.02 ± 0.01	0.06 ± 0.02	−0.03 ± 0.01

^a^ Averaged over all water molecules in Equations ((24), (25), (26)).

As shown in Table 4, metal ions are chemically hard, unwilling to share their electrons with water ($\Delta {\bar{E}}_{A\to 1\text{water}}^{\mathrm{CT}}$ and $\Delta {\bar{E}}_{A\to 2\text{waters}}^{\mathrm{CT}}$ are negligible), but they eagerly accept electrons from water ($\Delta {\bar{E}}_{1\text{water}\to A}^{\mathrm{CT}}$ and $\Delta {\bar{E}}_{2\text{waters}\to A}^{\mathrm{CT}}$ are large). Halide ions readily donate electrons to water ($\Delta {\bar{E}}_{A\to 1\text{water}}^{\mathrm{CT}}$ and $\Delta {\bar{E}}_{A\to 2\text{waters}}^{\mathrm{CT}}$ are substantial). The pairwise CT is roughly of the same magnitude for all water molecules (small standard deviations). For three‐body CT, larger standard deviations indicate that there is a variation caused by the relative orientation of 2 water molecules in trimers. The three‐body cross terms ($\Delta {\bar{E}}_{A\text{water}\to \text{water}}^{\mathrm{CT}}$ and $\Delta {\bar{E}}_{\text{water}\to A\text{water}}^{\mathrm{CT}}$) are substantial only for +2 and + 3 ions.

**FIGURE 4:**
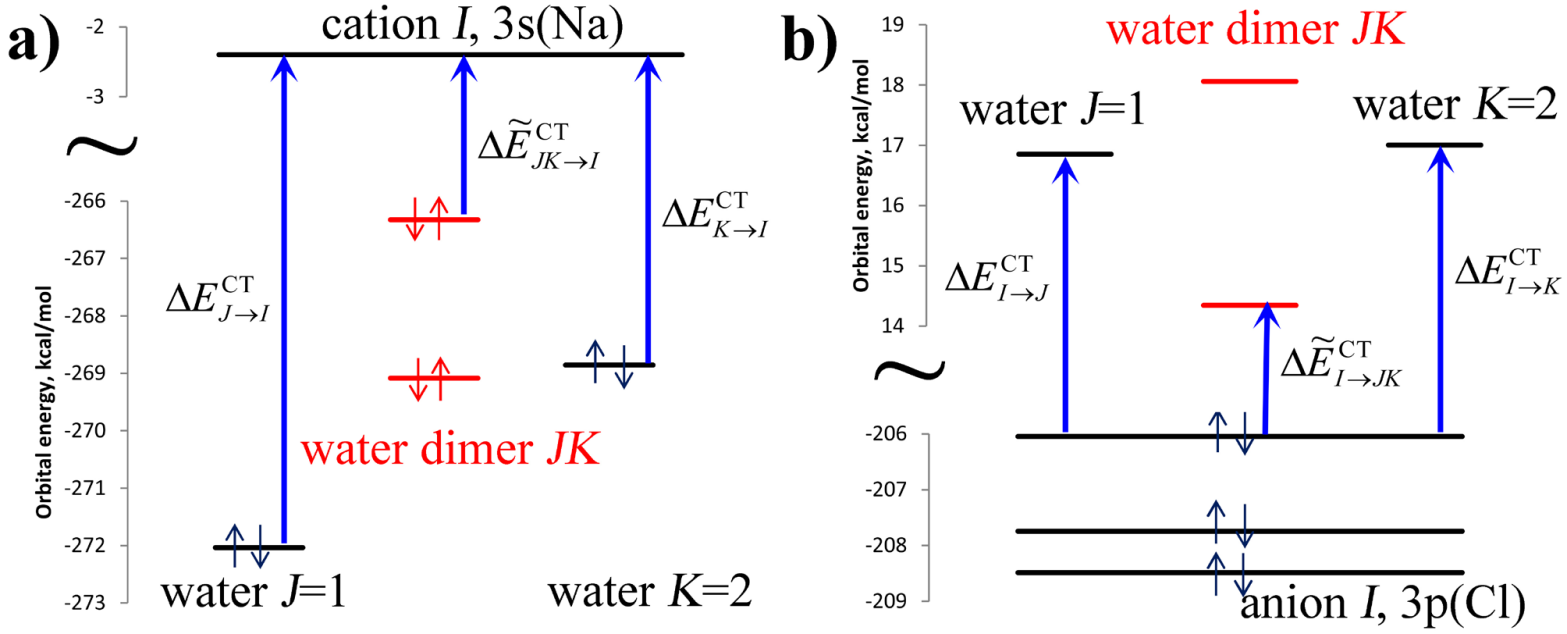
Orbital diagram illustrating ion‐water CT in (a) cation (Na^+^) (b) anion (Cl^−^). Orbital energies are taken from actual calculations for an arbitrarily selected pair of water molecules. Blue arrows representing CT show only a single frontier orbital pair whereas in reality all orbitals contribute.

A schematic representation of CT in Figure 4 shows a selection of frontier orbitals. For both cations and anions, the bulk CT in a trimer *IJK* $\Delta {\tilde{E}}_{I\to \mathrm{JK}}^{\mathrm{CT}}$ is attractive, because the coupling of monomer orbitals in dimer *JK* decreases the gap between occupied and virtual orbitals, promoting charge transfer as reflected in the bulk values that combine two and three‐body effects (as noted above, purely three‐body effects $\Delta E_{I\to \mathrm{JK}}^{\mathrm{CT}}$ can be both positive and negative).

CT in halides is enhanced by the triple‐degenerate filled valence p orbital, split in the field of the complex. In contrast, CT in monocations does not involve quasi‐degenerate frontier orbitals, and it is much smaller than in halides (Table 4). In addition, the gap between occupied and virtual orbitals is smaller in halides than in monocations, leading to a larger CT.

For ion *A* denoted as fragment *I* and water fragments numbered as *J* and *K*, the total CT can be obtained as

(27) $$\Delta E_{A\to \text{water}}^{\mathrm{CT}3}=\sum_{J=1}^{\text{water}}\Delta E_{I\to J}^{\mathrm{CT}}+\sum_{J>K}^{\text{water}}\Delta E_{I\to \mathrm{JK}}^{\mathrm{CT}}$$

(28) $$\Delta E_{\text{water}\to A}^{\mathrm{CT}3}=\sum_{J=1}^{\text{water}}\Delta E_{J\to I}^{\mathrm{CT}}+\sum_{J>K}^{\text{water}}\Delta E_{\mathrm{JK}\to I}^{\mathrm{CT}}$$

The total values of charge transfer in Equations (27) and (28) can be compared with reference (ref) values, which are obtained from a separate calculation where all water molecules are assigned into one merged fragment *W*, and the ion is left as a separate fragment (2 fragments total).

(29) $$\Delta E_{A\to \text{water}}^{\text{CTref}}=\Delta E_{I\to W}^{\mathrm{CT}}$$

(30) $$\Delta E_{\text{water}\to A}^{\text{CTref}}=\Delta E_{W\to I}^{\mathrm{CT}}$$

The comparison in Table 5 serves as an accuracy check of the many‐body expansion, as the reference values are considered exact (in the PL state picture), including CT up to 7‐body level in the set of 7 fragments. CT3 values are quite accurate, whereas two‐body CT2 values for +2 and +3 ions are underestimated relative to the reference (overestimated for Be^2+^).

**TABLE 5:** Total many‐body charge transfer energies (kcal/mol) in *A*(H_2_O)_6_, DFT/6–311++G**/SMD.

*A*	Charge transfer from ion to water			Charge transfer from water to ion		
	$\Delta E_{A\to \text{water}}^{\mathrm{CT}2}$	$\Delta E_{A\to \text{water}}^{\mathrm{CT}3}$	$\Delta E_{A\to \text{water}}^{\text{CTref}}$	$\Delta E_{\text{water}\to A}^{\mathrm{CT}2}$	$\Delta E_{\text{water}\to A}^{\mathrm{CT}3}$	$\Delta E_{\text{water}\to A}^{\text{CTref}}$
Li^+^	−0.1	−0.1	−0.1	−7.5	−14.8	−10.6
Na^+^	−0.3	−0.4	−0.4	−2.5	−5.6	−5.0
K^+^	−0.4	−0.4	−0.4	−5.2	−6.4	−6.2
Be^2+^	0.0	0.0	−0.1	−64.1	−55.8	−55.1
Mg^2+^	−0.2	−0.3	−0.3	−18.6	−33.8	−33.6
Ca^2+^	−0.7	−0.6	−0.6	−38.4	−70.0	−69.5
Al^3+^	−0.1	−0.3	−0.3	−94.9	−143.9	−147.2
F^−^	−20.7	−15.4	−16.6	−0.5	−0.8	−0.9
Cl^−^	−19.5	−18.8	−19.1	−0.9	−1.2	−1.1
Br^−^	−17.5	−18.4	−18.1	−1.1	−1.4	−1.3

**FIGURE 5:**
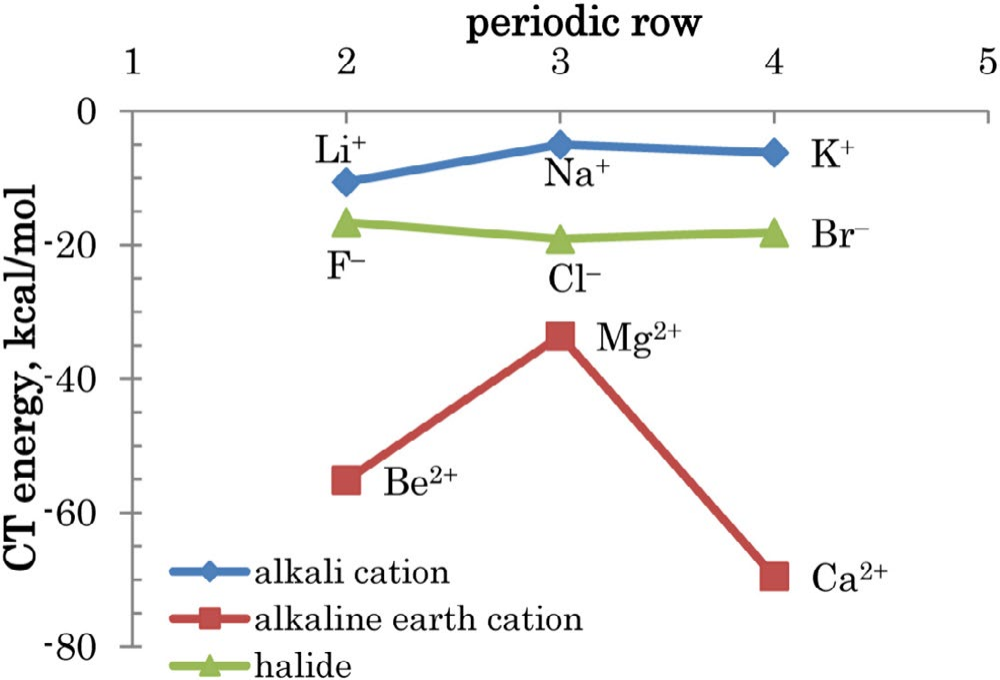
Total charge transfer energies $\Delta E_{\text{water}\to A}^{\text{CTref}}$ for cations and $\Delta E_{A\to \text{water}}^{\text{CTref}}$ for anions.

The trends of CT are shown in Figure 5. For cations, when going down the periodic table, valence unoccupied MOs are localized further away from the nucleus. This decreases the stabilization from populating these states by CT; however, the overlap with occupied orbitals of water increases. The combination of these opposite trends, with the additional factor of the molecular geometry (i.e., affected not only by CT but by the total binding), leads to a nonmonotonic dependence, with a maximum in the middle (the third row). For anions, there is a weak dependence of CT on the periodic row (with a barely visible minimum for the third row as well), attributed to a nearly constant ability of water to attract electrons, although heavier halides may be more willing to share them.

**FIGURE 6:**
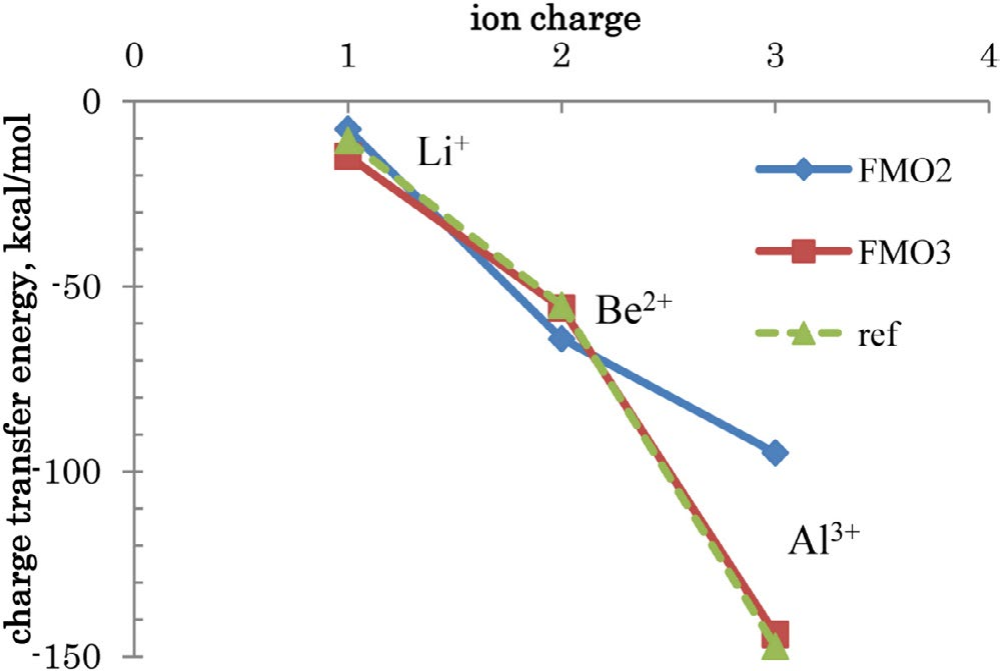
Water‐ion charge transfer energy $\Delta E_{\text{water}\to A}^{\text{CTref}}$ as a function of the ion charge.

Many‐body effects for a series of ions are shown in Figure 6. Up to the charge of +2, both FMO2 and FMO3 perform quite well, but for +3, three‐body effects are very large and only FMO3 reproduces the reference values.

### 4.5 Comparison of DFT and MP2 for Solvated Ions

**FIGURE 7:**
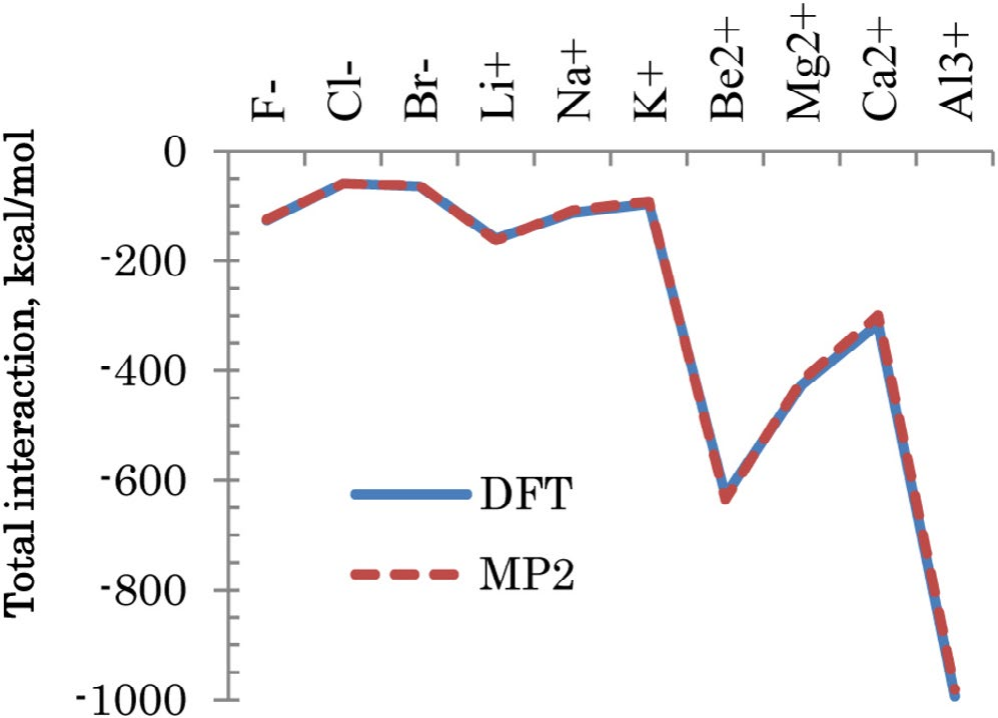
Total interaction energies ΔE of ions with water.

In the comparison of DFT and MP2, the setup with 2 fragments (ion and all water) is used. The results are shown in Figure 7. At this scale, a little difference is seen, although individual values differ as much as 16 kcal/mol for di‐ and tri‐cations.

**FIGURE 8:**
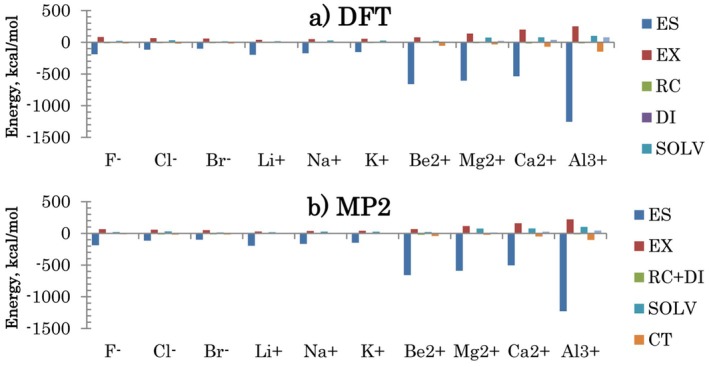
Interaction energy components in ion‐water complexes.

Individual components are compared in Figure 8. The interactions are dominated by ES, partially offset by the solvent screening SOLV. Drawn by the strong electrostatics, ions and water repel each other as expressed by the EX term. Overall, DFT and MP2 have a similar set of components.

Halide and alkali ions have comparable values of interaction energies. In each family (alkali, alkaline earth, and halide), there is a trend for the total interaction energies to decrease with the periodic row, attributed to the shielding of valence electrons by the chemical core, weakening the binding of ions to water.

**FIGURE 9:**
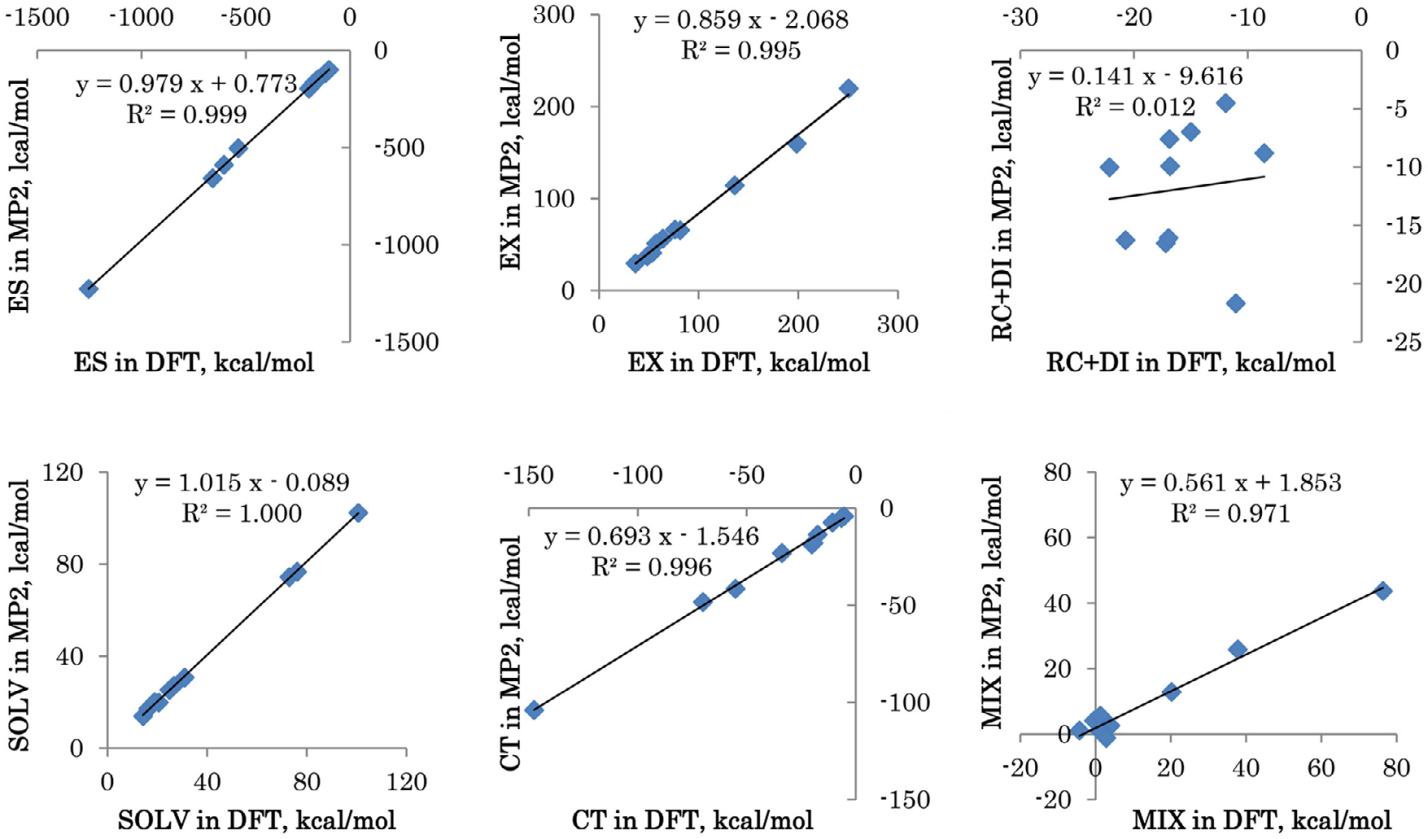
Comparison of DFT and MP2 components for solvated ions (all 10 ions are taken as 1 set).

Individual components are compared in Figure **9**. There is a very high correlation with coefficients *R* ^2^ close to 1 for all terms except for the electron correlation RC + DI. In DFT, DI is obtained with an empirical model D3(BJ), which may not be able to capture strong charge delocalization effects on dispersion in solvated ions. CT and MIX are much larger in DFT than in MP2, related to the smaller gap between occupied and virtual orbitals. The MIX term features a much worse correlation for monoions (the swarm of points near 0), where absolute values are small and deviations are more pronounced. EX is somewhat smaller in MP2 (by about 14%).

**FIGURE 10:**
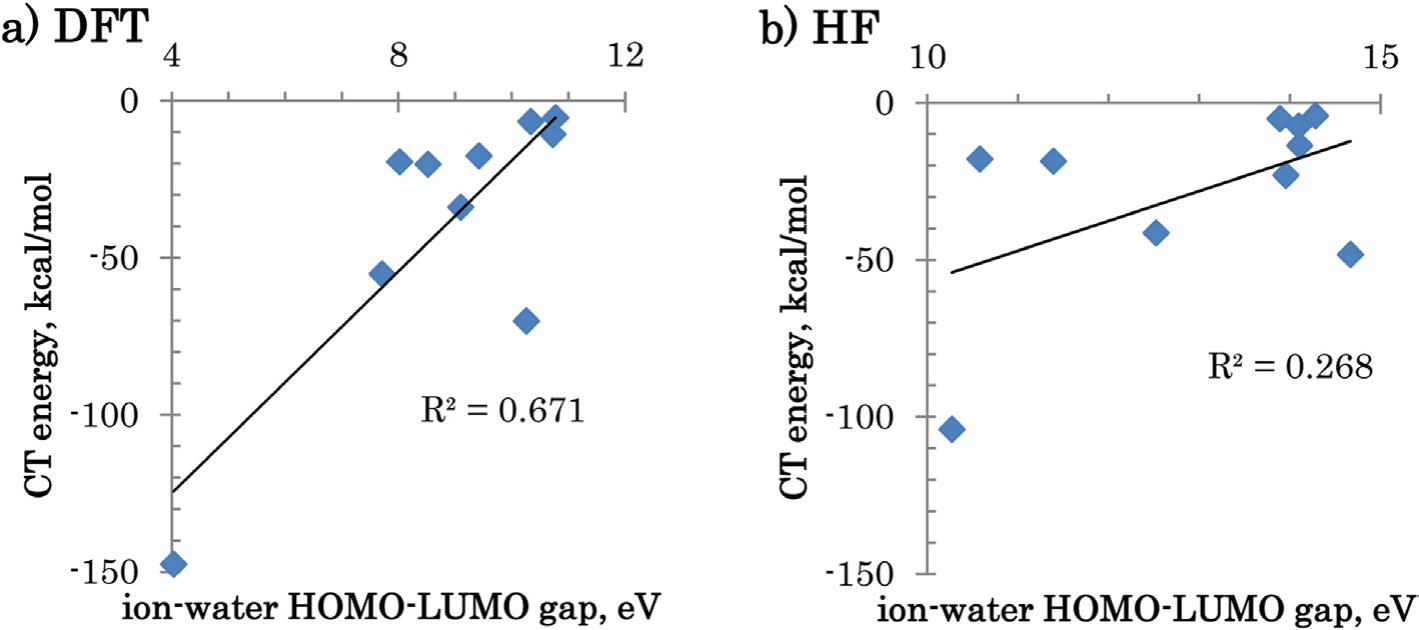
CT energy ($\Delta E_{\text{water}\to A}^{\text{CTref}}$ for cations or $\Delta E_{A\to \text{water}}^{\text{CTref}}$ for anions) vs. ion‐water HOMO‐LUMO gap, (a) DFT and (b) HF (all 10 ions are taken as 1 set).

There is a weak correlation of the ion‐water HOMO‐LUMO gap with the CT energy, plotted for 2‐fragment calculations in Figure 10, with a somewhat stronger correlation for DFT (apparently due to the Al^3+^ outlier that increases *R* ^2^). This means that it is not appropriate to think that CT occurs only between HOMO and LUMO (which in a way warns against using those descriptors in conceptual DFT that focus on frontier orbitals, for fragments where multiple MOs may be important).

As shown in Figure S1, within each ion family, only halides have a high correlation to the gap. This also holds for DFT, with very little correlation for alkali and alkaline earth ions. The reason why halides have the correlation is that CT is from a single nearly degenerate *n*p occupied orbital, whereas for cations CT involves multiple orbitals.

## 5 Conclusions

In this work, a methodology for defining charge transfer energies in the fragment molecular orbital method has been developed at the two‐and three‐body level (it may be noted that there is an alternative way [50] of computing two‐body values in FMO based on configuration interaction). The two‐and three‐body CT scheme, proposed in this work, may be extended in future to four‐body [93, 94] for higher accuracy.

It has been shown that the CT in FMO exactly agrees with KM‐EDA for 2 fragments provided that the same type of the reference state is used. The MIX term is strongly affected by the embedding type. The CT in hydrogen bonds is dominated by the charge transfer of the electron pair, but there is also a non‐negligible backward donation. The CT of two hydrogen bonds per fragment pair found in β‐turns of polypeptides is decoupled with the directional scheme developed in this work.

DFT is found to have a larger CT energy than HF, attributed to a smaller gap. Individual three‐body terms are fairly small in water and monoions, but there are many of them, and they may add up to a substantial value. For dications and trications, three‐body CT terms are large. The trends in the CT energy of ions across the periodic table have been revealed, featuring an extremum for the third row.

A high correlation is found for DFT and MP2 interaction energies, except for the electron correlation term RC + DI. The CT and MIX terms are much larger in DFT compared to MP2 because of a smaller gap between occupied and virtual orbitals.

KM‐EDA and other decomposition analyses have fostered the development of polarizable force fields [95, 96, 97, 98]. FMO [99, 100] and other fragment‐based methods [101] have been used for refining atomic charges in force fields. Incorporating charge transfer into force fields is an important route for their improvement, and it is hoped that the development in this work may be useful in this regard.

## Supporting information


Data S1.

## Data Availability

The data that support the findings of this study are available from the corresponding author upon reasonable request.
