# Supplementary material for: Analyzing Many‐Body Charge Transfer Effects With the Fragment Molecular Orbital Method
Source: J Comput Chem. 2025 May 15;46(13):e70128. doi: 10.1002/jcc.70128 (PMC12079640; doi:10.1002/jcc.70128)
Supplement: Supplementary file 1 — Data S1. [file JCC-46-0-s001.pdf]

Supporting Information for

# Analyzing many-body charge transfer effects with the fragment molecular orbital method

*Dmitri G. Fedorov*

Materials DX Research Center, National Institute of Advanced Industrial Science and Technology (AIST), Central 2, Umezono 1-1-1, Tsukuba, 305-8568, Japan.

## 1. Methodology

### 1.1 Projection operator

For dimer *IJ*, its Fock matrix $\mathbf{F}$ in the AO basis is made of contributions from the projection operator $\mathbf{P}$ and the rest of terms $\mathbf{F}'$, $\mathbf{F} = \mathbf{F}' + \mathbf{P}$. CT energy components are obtained by solving constrained HF equations for the Fock matrix in the basis of monomer MOs (in which one can enforce the allowed mixing required for EDA).

The AO to MO transformation of the projection matrix $\mathbf{P}$ is

$$\widetilde{\mathbf{P}} = \mathbf{C}^{\dagger}\mathbf{P}\mathbf{C} \qquad \text{(S1)}$$

where $\mathbf{C}$ is the combined matrix of the MOs of isolated monomers *I* and *J*, stored as blocks in a matrix for dimer *IJ*, padded by zeros.

Using the direct sum form of the projection operator (see main text) and the block form of $\mathbf{C}$,

$$\mathbf{P}=\begin{bmatrix}\mathbf{C}^{I\dagger} & 0\\ 0 & \mathbf{C}^{J\dagger}\end{bmatrix}\begin{bmatrix}\mathbf{P}^{I} & 0\\ 0 & \mathbf{P}^{J}\end{bmatrix}\begin{bmatrix}\mathbf{C}^{I} & 0\\ 0 & \mathbf{C}^{J}\end{bmatrix}=\begin{bmatrix}\mathbf{C}^{I\dagger}\mathbf{P}^{I}\mathbf{C}^{I} & 0\\ 0 & \mathbf{C}^{J\dagger}\mathbf{P}^{J}\mathbf{C}^{J}\end{bmatrix} \tag{S2}$$

Thus it can be seen that the block structure of both **C** and **P** results in the decoupled application of the projection operator of *I* on orbitals of *I* and likewise for *J*. Because MOs of each monomer are obtained in unconstrained calculations with the respective monomer projection operator, the orbitals in *I* or *J* are properly relaxed with respect to its own projection.

**1.2 Justification of the polarization term in PL0**

The PIE coupling term is

$$\Delta E_{IJK} = \Delta E'_{IJK} + \Delta E^{\text{CT·ES}}_{IJK} \tag{S3}$$

where

$$\Delta E'_{IJK} = E'_{IJK} - E'_{I} - E'_{J} - E'_{K} - \Delta E'_{IJ} - \Delta E'_{IK} - \Delta E'_{JK} \tag{S4}$$

and

$$\Delta E^{\text{CT·ES}}_{IJK} = Tr\left(\Delta\mathbf{D}^{IJK}\mathbf{V}^{IJK}\right) - Tr\left(\Delta\mathbf{D}^{IJ}\mathbf{V}^{IJ}\right) - Tr\left(\Delta\mathbf{D}^{IK}\mathbf{V}^{IK}\right) - Tr\left(\Delta\mathbf{D}^{JK}\mathbf{V}^{JK}\right) \tag{S5}$$

The $E'_{IJK} - E'_{I} - E'_{J} - E'_{K}$ term in eq S4 gives rise to one set of polarization energies, $\Delta E^{\text{POL0}}_{I} + \Delta E^{\text{POL0}}_{J} + \Delta E^{\text{POL0}}_{K}$ (because isolated monomers *I*, *J*, and *K* are polarized in the trimer), whereas the remaining 3 terms, $-\Delta E'_{IJ} - \Delta E'_{IK} - \Delta E'_{JK}$ contribute $-\left(\Delta E^{\text{POL0}}_{IJ} + \Delta E^{\text{POL0}}_{IK} + \Delta E^{\text{POL0}}_{JK}\right)$. So that the total polarization in *IJK* is

$$\Delta E_{IJK}^{\mathrm{POL0}} = \Delta E_{I}^{\mathrm{POL0}} + \Delta E_{J}^{\mathrm{POL0}} + \Delta E_{K}^{\mathrm{POL0}} - \left(\Delta E_{IJ}^{\mathrm{POL0}} + \Delta E_{IK}^{\mathrm{POL0}} + \Delta E_{JK}^{\mathrm{POL0}}\right) \quad \text{(S6)}$$

In eq S5, $\Delta\mathbf{D}^{X}$ is the density transfer matrix in *X*, and $\mathbf{V}^{X}$ is the matrix of the embedding potential (*X*=*IJ* or *IJK*).

## 2. Results

Table S1. Comparison of polarization (POL) and binding energies (BE), (kcal/mol) vs full KM-EDA for $(H_2O)_2$ at the level of HF/6-311++G**.

| Method | ESP[a] | POL | BE |
|---|---|---|---|
| EDA2/PL0 | damp-pc | -0.333 | -4.124 |
| EDA2/PL0 | density | -1.639 | -4.124 |
| EDA2/PL | damp-pc | -0.529 | -4.124 |
| EDA2/PL | density | -1.751 | -4.124 |
| KM-EDA | density | -1.751 | -4.124 |

Table S2. Comparison between KM and RVS decompositions for $(H_2O)_2$ at the level of HF/6-311++G**.

| method | ES | POL | EX | CT | MIX[a] | total |
|---|---|---|---|---|---|---|
| KM-EDA | -12.60 | -1.75 | 11.86 | -2.81 | 1.17 | -4.12 |
| RVS | -12.59 | -1.41 | 11.86 | -1.41 | -0.57 | -4.12 |

[a] The difference between the total interaction and other components.

Table S3. The effect of the AO type for energy components (kcal/mol) in EDA2 for $(H_2O)_2$ at the level of HF/6-311++G**.[a]

| Method | AO | ES | EX | CT($I\rightarrow J$) | CT($J\rightarrow I$) | MIX |
|---|---|---|---|---|---|---|
| EDA2/PL0 | Cartesian | -12.599 | 11.862 | -0.781 | -2.025 | -0.248 |
| EDA2/PL0 | spherical | -12.602 | 11.868 | -0.777 | -2.028 | -0.251 |
| EDA2/PL | Cartesian | -13.519 | 11.366 | -0.473 | -2.018 | 0.129 |
| EDA2/PL | spherical | -13.517 | 11.374 | -0.471 | -2.021 | 0.128 |

[a] Using the damped point charges (damp-pc) ESP.

Table S4. $\Delta E_{AI}^{\mathrm{CT}}$ values (kcal/mol) for charge transfer between ion *A* and water molecule *I* in $A(H_2O)_6$, DFT/6-311++G**.

| *I* | $Li^+$ | $Na^+$ | $K^+$ | $Be^{2+}$ | $Mg^{2+}$ | $Ca^{2+}$ | $Al^{3+}$ | $F^-$ | $Cl^-$ | $Br^-$ |
|---|---|---|---|---|---|---|---|---|---|---|
| 1 | -1.14 | -0.50 | -0.82 | -10.11 | -3.12 | -6.32 | -15.77 | -3.18 | -3.34 | -3.08 |
| 2 | -1.50 | -0.40 | -0.85 | -11.17 | -3.12 | -6.37 | -15.89 | -3.18 | -4.09 | -3.81 |
| 3 | -1.27 | -0.59 | -1.04 | -11.34 | -3.21 | -6.88 | -15.94 | -3.84 | -3.12 | -2.79 |
| 4 | -1.45 | -0.40 | -1.02 | -10.61 | -3.04 | -6.57 | -16.04 | -3.86 | -3.38 | -2.89 |
| 5 | -1.07 | -0.28 | -0.96 | -10.69 | -3.13 | -6.59 | -15.79 | -3.54 | -3.32 | -3.12 |
| 6 | -1.19 | -0.55 | -0.93 | -10.18 | -3.17 | -6.31 | -15.65 | -3.54 | -3.14 | -2.97 |

Table S5. Comparison of CAM-B3LYP and M06 reference charge transfer energies (kcal/mol) $\Delta E_{\mathrm{water}\rightarrow A}^{\mathrm{CT}}$ for cations and $\Delta E_{A\rightarrow \mathrm{water}}^{\mathrm{CT}}$ for ions *A* in $A(H_2O)_6$, 6-311++G**.

| *A* | CAM-B3LYP | M06 |
|---|---|---|
| $Li^+$ | -10.6 | -9.7 |
| $Na^+$ | -5.0 | -6.4 |
| $K^+$ | -6.2 | -5.1 |
| $Be^{2+}$ | -55.1 | -48.0 |
| $Mg^{2+}$ | -33.6 | -32.3 |
| $Ca^{2+}$ | -69.5 | -66.0 |
| $Al^{3+}$ | -147.2 | -138.5 |
| $F^-$ | -16.6 | -15.0 |
| $Cl^-$ | -19.1 | -17.1 |
| $Br^-$ | -18.1 | -16.5 |

Table S6. Atomic charges $Q_A$ of ions $A$ in $A(H_2O)_6$, DFT/6-311++G**.

| $A$ | Mulliken | Stone[a] |
|---|---|---|
| $Li^+$ | 0.52 | 0.92 |
| $Na^+$ | 0.77 | 0.90 |
| $K^+$ | 0.96 | 0.90 |
| $Be^{2+}$ | 0.73 | 1.78 |
| $Mg^{2+}$ | 1.18 | 1.78 |
| $Ca^{2+}$ | 1.44 | 1.72 |
| $Al^{3+}$ | 2.13 | 1.93 |
| $F^-$ | -0.68 | -1.02 |
| $Cl^-$ | -0.74 | -0.85 |
| $Br^-$ | -0.71 | -0.84 |

[a] With the diffuse exponents below 0.3 integrated numerically.

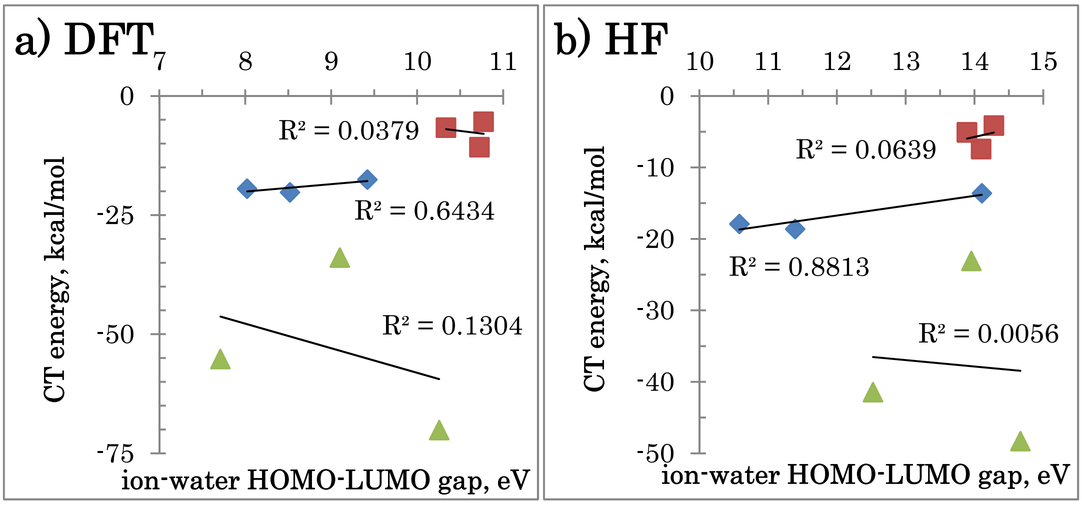


Figure S1. CT energy $\Delta E^{\mathrm{CTref}}_{\mathrm{water},A}$ vs ion-water HOMO-LUMO gap, (a) DFT and (b) HF shown separately for alkali (red), alkaline earth (green) and halides (blue).
